# Numerical Modelling of Pulsed Laser Surface Processing of Polymer Composites

**DOI:** 10.3390/ma19030607

**Published:** 2026-02-04

**Authors:** Krzysztof Szabliński, Krzysztof Moraczewski

**Affiliations:** Department of Polymer Materials Engineering, Faculty of Materials Engineering, Kazimierz Wielki University, Chodkiewicza 30, 85-064 Bydgoszcz, Poland

**Keywords:** numerical methods, material modelling, advanced materials, engineered materials

## Abstract

Filled-polymer coatings enable functional surfaces for selective metallisation, wetting control and local conductivity, but pulsed-laser texturing is often limited by process non-uniformity caused by scan kinematics and plume shielding. Here, we develop a three-tier numerical workflow for nanosecond pulsed-laser surface treatment of a thermoplastic coating containing glass microspheres (baseline case: PLA matrix with V_f_ = 0.20; spheres represented via an effective optical transport model). Tier 1 predicts spatially resolved ablation depth under raster scanning, using an incubation law and regime switching (no-removal/melt-limited/logarithmic ablation/blow-off) coupled to a dynamic shielding factor. Tier 2 computes the 1D transient (pulse-averaged) temperature field and the thickness of the thermally softened layer. Tier 3 models post-pulse capillary redistribution of the softened layer to estimate groove reshaping. The simulations show that scan overlap and shielding dynamics dominate groove homogeneity more strongly than average power alone: under identical average power, variations in local pulse count and shielding lead to significant changes in depth statistics and regime fractions. The workflow produces quantitative maps and summary metrics (mean depth, P5–P95 range, uniformity index and regime fractions) and demonstrates how controlled reflow can smooth peaks while preserving groove depth. These results provide a predictive tool for laser parameter selection and process optimisation prior to experimental trials.

## 1. Introduction

Pulsed laser micromachining is an established approach to precision surface texturing and pattern transfer in polymers and polymer composites, enabling functional micro-features over centimetre-scale areas with limited collateral damage [1,2,3]. In practical high-throughput operation, the surface is not shaped by an isolated pulse. Instead, dense pulse overlap at high repetition rates means that incubation, inter-pulse heat accumulation, and plume–beam interactions strongly condition the morphology and yield. These phenomena are extensively discussed in the ultrashort- and short-pulse literature and provide a useful conceptual framework for nanosecond raster scanning as well [4,5,6]. Similar cumulative effects (incubation, heat accumulation, and plume/plasma shielding) have been reported in both ultrafast high-repetition-rate processing and nanosecond scanning regimes; here, we focus on the nanosecond-class Nd:YAG case.

In this work, we consider a high-repetition-rate, 1064 nm, nanosecond-class Q-switched Nd:YAG regime (typically ≈ 60 kHz, ≈0.2 mJ per pulse, with scan speeds on the order of 10^3^ mm s^−1^), in which the interpulse spacing along the scan path becomes comparable to the thermal diffusion length. The pulse duration is ≈80 ns, i.e., nanosecond-class rather than femtosecond. Under these conditions, heat accumulation, incubation-driven threshold evolution, and transient plume/plasma shielding are primary process determinants, rather than secondary corrections. Consequently, process development still relies heavily on empirical design-of-experiments, motivating reduced-order, physics-grounded models that are predictive yet computationally efficient. Recent reviews and modelling studies emphasise the need to integrate optical absorption, multi-pulse damage/threshold kinetics, scanning strategy, and thermal-hydrodynamic response within a single workflow rather than treating them separately [4,5,6,7].

In this study, the object is a thermoplastic polymer coating (PLA as the baseline matrix) containing micron-scale glass microspheres (solid spheres, optionally coated with PDA/Ag for downstream electroless metallisation). The present manuscript focuses on laser-induced topography formation and therefore represents the composite through (i) an effective optical transport description (absorption + scattering) and (ii) bulk thermophysical properties; particle exposure is discussed as a process outcome that is relevant for subsequent metallisation.

Filled-polymer coatings add an additional layer of complexity and practical value: embedded particles reshape optical and thermal transport and, after laser exposure, can act as functional surface features. Beyond optical and thermal transport, embedded fillers can also modify electrical charge transport and related functional responses; in particular, conductive or metal-seeded fillers may create charge pathways whose state can change after laser processing. In our case, the PDA/Ag coating is introduced as a catalytic seed layer for subsequent electroless deposition, while the present model captures the primary optical–thermal consequences that are relevant to groove formation [8]. For optics, effective-medium approaches such as Maxwell Garnett or Bruggeman mixing provide first-order estimates of composite permittivity, while their limitations—especially near resonances or at large inclusion contrast—are well documented [9,10,11,12]. Temperature-dependent dielectric functions further modify absorption during rapid heating. These aspects have been revisited for heterogeneous media and, for metals and semiconductors, quantified using up-to-date optical data, together with cautions regarding simple mixing rules [13,14,15,16]. Scattering by the particles themselves can be described by Mie theory, supplying absorption and scattering efficiencies that map into transport coefficients for volumetric source terms. Beyond optics, exposing metallisable or PDA/Ag-coated microspheres at the surface after scanning can seed selective electroless metallisation, tune local wettability/adhesion, and enable patterned conductive features on lightweight polymer substrates. This links laser-driven morphology control directly to applications in metallisation, EMI shielding, bonding, and interfacial engineering.

Laser-matter response under dense multi-pulse irradiation spans regimes from sub-threshold heating through melt formation, logarithmic ablation above threshold, and, at sufficiently high deposited energy densities, blow-off accompanied by strong plume formation. The sequence and thresholds depend on pulse count through incubation, on the evolving thermal background set by scanning (heat accumulation), and on local attenuation of subsequent pulses by the ablation plume (plume/plasma shielding). Contemporary summaries detail these regime transitions and their dynamics under high-repetition-rate pulsed irradiation across materials and pulse durations, including the nanosecond regime that is relevant to industrial texturing [17,18,19,20].

High-repetition-rate nanosecond raster scanning therefore combines cumulative heating, incubation of the ablation threshold under repeated irradiation, and transient attenuation of the incoming beam by the evolving plume/plasma above the surface (plume shielding) [21,22,23,24,25]. These coupled effects can strongly modulate the effective fluence delivered to each point along the scan path and thus influence groove depth, rim formation, and feature rounding. Accordingly, the present model targets the nanosecond regime and explicitly couples scan kinematics with incubation and a reduced-order plume shielding factor.

At high repetition rates, the inter-pulse spacing approaches the thermal diffusion length and heat accumulation becomes a first-order design parameter. Scaling analyses and measurements show that the accumulation index depends on thermal diffusivity, repetition rate, and scan speed; practically, the same pulse energy can yield different peak temperatures and morphologies as overlap is varied [4,5,6,7,18,19,26]. In parallel, laser-induced plumes and nascent plasmas can attenuate the incident beam (“plume/plasma shielding”), reducing the effective fluence and modifying crater growth under repeated irradiation effects, observed experimentally and captured by simplified attenuation models [19,20,22,23]. Multipulse incubation lowers the effective ablation threshold with a pulse number toward an asymptote; empirical power-law forms remain widely used in process modelling [18,19].

Whenever transient melting occurs—which is common in polymers and many composites once cumulative heating pushes the matrix into the softening/melting range—the final surface is not a frozen “crater profile” from the last pulse. Instead, it reflects capillary-driven levelling, opposed by viscosity and solidification. This post-solidification shaping stage is commonly represented using thin-film (lubrication) hydrodynamics, which has a mature theoretical basis and multiple validations in laser-reflow contexts; it is therefore natural to include such a module when predicting post-process roughness, rim width, and feature rounding [27,28,29,30].

Here, we assemble these ingredients into a reduced-order, simulation-ready pipeline tailored to particle-loaded polymer coatings. The workflow combines: (i) composite optics via effective-medium mixing and Mie-informed transport; (ii) Beer–Lambert volumetric heating with temperature-dependent absorption and reflectance; (iii) a dynamic plume-shielding state variable that attenuates the local fluence between closely spaced pulses; (iv) an incubation law for the evolving threshold; (v) a multi-regime per-pulse depth increment spanning no removal, melting without ablation, logarithmic ablation, and blow-off; and (vi) a thin-film capillary reflow step, together with a thermoelastic surrogate for stress-coupled relief. We target raster strategies that are typical of industrial texturing and report regime maps, topography fields, and statistical descriptors that link directly to process controls (fluence, overlap, duty factor, and plume time constant). The intent is to provide a compact yet comprehensive model that is fast enough for design-space exploration while remaining faithful to the dominant physics highlighted in recent high-repetition-rate pulsed laser processing studies.

The novelty of the present work is the explicit coupling of realistic scan kinematics, incubation-driven threshold reduction, time-dependent plume/plasma shielding *S(t)*, temperature-dependent composite optics, melt-layer thickness prediction, and thin-film capillary reflow into a single reduced-order workflow for filled polymer coatings. For clarity, the main symbols and abbreviations are summarised in the Nomenclature section.

## 2. Materials and Methods

We simulated raster-scan laser processing of a rectangular-parallelepiped polymer coating, using a custom multi-tier numerical workflow implemented in GNU Octave. The heat source was a Gaussian, Q-switched Nd:YAG laser (*λ* = 1064 nm) operated at a repetition rate of *f* = 60 kHz and an average power of *P_avg_* = 12 W, corresponding to a pulse energy of *E_p_* ≈ $\frac{P_{avg}}{f}\approx \;$0.2 mJ. The pulse duration was in the nanosecond range (τp ≈ 80 ns; manufacturer specification), i.e., a nanosecond high-repetition-rate regime rather than isolated ultrashort single-pulse ablation. Unless stated otherwise, the nominal scan speed was *v_scan_* = 1.0 m·s^−1^ (≈10^3^ mm·s^−1^). The resulting along-track interpulse spacing is Δx = $\frac{v_{scan}}{f}$ ≈ 16.7 μm, i.e., on the order of tens of micrometres. Under these conditions, the interpulse spacing becomes comparable to the thermal diffusion length in the polymer on the inter-pulse timescale. Consequently, inter-pulse heat accumulation, incubation-driven threshold evolution, and plume/plasma shielding of subsequent pulses are treated explicitly as first-order effects.

The modelled system is a thermoplastic polymer coating (baseline: PLA) containing micron-scale glass microspheres. The coating thickness was set to *L_z_* = 0.5 mm and the simulated surface window to *L_x_ × L_y_* = 2 mm × 2 mm. The filler content is represented by the volume fraction *V_f_* (baseline *V_f_* = 0.20) and a sphere size distribution parameterised by the mean radius $\bar{r}$ = 25 μm and standard deviation *σ_r_* = 5 μm, used as input descriptors for the effective optical transport model (Table 1). When PDA/Ag-coated microspheres are considered, Ag is treated as a catalytic seed layer for subsequent electroless metallisation, rather than as a bulk reinforcing phase; the present workflow therefore focuses on laser-induced topography formation and the thermally affected layer that is relevant to melt-mediated reshaping.

In the thermo-optical tier, absorption was represented using Beer–Lambert volumetric attenuation with optical properties permitted to vary with temperature. Multi-pulse incubation and plume attenuation were incorporated as described in Section 2.7 and Section 2.8. The thermal conduction and thin-film hydrodynamics tiers were evaluated for representative cases reported in the Results, whereas the ablation-only tier was used for rapid parametric mapping of the (*P, v*) process window.

The solver outputs include spatial maps of pulses-per-pixel, effective ablation threshold, plume shielding factor, per-pulse and cumulative ablation depth, predicted melt-layer thickness, and post-reflow surface topography. For clarity, the workflow is organised into three tiers: (i) a kinematics-based ablation tier that converts scan delivery, incubation, and shielding into a cumulative depth field without explicitly solving heat flow; (ii) a transient 1D thermo-optical tier that resolves heat deposition and through-thickness diffusion to estimate melt-layer thickness; and (iii) a thin-film melt-flow tier that evolves the molten surface by capillary-driven levelling prior to freeze-in. These tiers correspond to the three classes of results reported in Section 3.

Table 1 summarises the numerical and material parameters used in the simulations, together with their physical meaning and units. Laser parameters (*λ*, *f_rep_*, *P_avg_*, *τ_p_*) follow manufacturer specifications for the employed Nd:YAG source, whereas scan parameters (*v_scan_*, hatch, turnaround dwell) reflect typical industrial raster strategies. Thermophysical properties (*ρ*, *c_p_*, *k*) were taken from the representative PLA literature ranges and treated as baseline values for sensitivity screening. Optical-transport proxies (*V_f_*, $\bar{r}$, *σ_r_*, *g* and scattering-strength coefficient) were selected to reproduce the expected trend of increased effective attenuation in microsphere-filled coatings. Incubation and shielding parameters (*F_1_*, *F_∞_*, *ξ*, *τ_sh_*, *s_max_*, *κ_sh_*) are explicitly treated as model parameters; they were chosen within the physically plausible ranges reported for multipulse nanosecond processing and are intended for subsequent calibration against profilometry/topography data.

### 2.1. Radius Distribution and Particle Count

For a prescribed filler volume fraction *ϕ* and a simulation box of size $L_{x}L_{y}L_{z}$, the number of spheres *N_s_* is obtained by matching the third moment ⟨*r*^3^⟩ of the chosen radius distribution to the target filler volume (Equation (1)). Specifically,

(1) $$N_{s}\approx max\left(1,\;\left\lfloor \frac{\phi \;L_{x}L_{y}L_{z}}{\frac{4}{3}\pi {\langle r}^{3}\rangle }\right\rfloor \right)$$

where ⟨*r*^3^⟩ is evaluated from the prescribed distribution (e.g., normal or log-normal) and $N_{s}$ is rounded to the nearest integer. This step defines the initial stochastic microstructure used by the optical-transport tier.

### 2.2. Packing (RSA + Elastic Relaxation)

Particles are first placed using random sequential adsorption (RSA) under periodic boundary conditions (PBC), and subsequently relaxed using a damped repulsive penalty to eliminate overlaps while preserving the target *ϕ*.

(2) $$∥xi-xj∥PBC\;\geq r_{i}\;+r_{j}-\varepsilon$$

with *ε* denoting a small numerical tolerance (Equation (2)). If needed, a simple volume-fraction control loop rescales all radii uniformly to keep the final filler content within tolerance. This RSA + relaxation procedure is widely used in stochastic microstructure generation and yields realistic nearest-neighbour statistics without imposing artificial ordering.

### 2.3. Microstructure Statistics

To verify that the packing is spatially homogeneous and that the prescribed *φ* is met, we report (i) the pair-correlation function *g(r)* and (ii) the nearest-neighbour distance distribution for the final relaxed configuration (Equations (3) and (4)). The reported *g(r)* is computed from the binned pair counts and normalised by the shell volume and the particle number density *ρ*.

(3) $$g\left(r_{k}\right)=\frac{n_{k}}{4\pi \;r_{k}^{2}\Delta r\;\rho \;N_{s}}$$

(4) $$\rho =\frac{N_{s}}{L_{x}L_{y}L_{z}}$$

### 2.4. Effective Properties (Auxiliary)

The microsphere-filled polymer is treated as an isotropic two-phase composite, consisting of a polymer matrix (subscript *m*) and spherical glass inclusions (subscript *f*) with volume fraction *φ*. Effective properties are denoted by the subscript *eff*.

Thermal conductivity is estimated using two classical effective-medium closures. The Maxwell Garnett (MG) expression is used as a first-order estimate for dispersed spherical inclusions in a continuous matrix (Equation (5)).

(5) $$k_{eff}=k_{m}\;\frac{k_{f}+2k_{m}+2\varphi (k_{f}-k_{m})}{k_{f}+2k_{m\;}-\varphi (k_{f\;}-k_{m})}$$

As an alternative, the symmetric Bruggeman self-consistent relation is used when inclusion–inclusion interactions are expected to be stronger (Equation (6)).

(6) $$\varphi \frac{k_{f}-k_{eff}}{k_{f}+2k_{eff}}+\left(1-\varphi \right)\frac{k_{m}-k_{eff}}{k_{m}+2k_{eff}}=0$$

Here, *k* is the thermal conductivity; *k_m_* and *k_f_* are the matrix and filler conductivities, respectively, and *φ* is the filler volume fraction (0 ≤ *φ* < 1).

For elastic properties, we employ a Mori–Tanaka scheme for spherical inclusions, formulated in terms of bulk modulus *K* and shear modulus *G*. With *K_m_*, *G_m_* and *K_f_*, *G_f_* denoting matrix and filler moduli, the effective bulk and shear moduli are given by Equations (7)–(9):

(7) $$K_{eff}=K_{m}+\frac{\varphi \left(K_{f}-K_{m}\right)}{1+\left(1-\varphi \right)\frac{K_{f}-K_{m}}{K_{m}+\frac{4}{3}G_{m}}}$$

(8) $$G_{eff}=G_{m}+\frac{\varphi \left(G_{f}-G_{m}\right)}{1+\left(1-\varphi \right)\frac{G_{f}-G_{m}}{G_{m}+f\_s}}$$

where

(9) $$f_{s}=G_{m}\frac{9K_{m}+8G_{m}}{6\left(K_{m}+2G_{m}\right)}$$

and the effective Young’s modulus *E_eff_* and Poisson’s ratio *ν_eff_* are obtained from Equations (10) and (11):

(10) $$E_{eff}=\frac{9K_{eff}\;G_{eff}}{3K_{eff}\;+\;G_{eff}}$$

(11) $$\nu_{eff}=\frac{3K_{eff}-2G_{eff}}{2\left(3K_{eff}+G_{eff}\right)}$$

The volume fraction *φ* refers to the microsphere fraction in the solid composite (not to porosity).

### 2.5. Optics and Heat Source (1064 nm)

Composite permittivity $\varepsilon_{eff}$ and optical indices $n_{eff},k_{eff}$ are obtained from effective-medium mixing of the PLA matrix and PDA/Ag-coated glass microspheres, using Maxwell Garnett/Bruggeman relations (Equations (12) and (13)) with *ε = (n + i k)*^2^. This provides a standard first-order treatment for particulate composites; its known limitations at high index contrast or near resonances are acknowledged in Section 4 [9,10,11,12].

(12) $$\varepsilon_{eff}=\varepsilon_{m}\frac{\varepsilon_{f}+2\varepsilon_{m}+2\varphi \left(\varepsilon_{f}-\varepsilon_{m}\right)}{\varepsilon_{f}+2\varepsilon_{m}-\varphi \left(\varepsilon_{f}-\varepsilon_{m}\right)}$$

(13) $$n_{eff}+ik_{eff}=\sqrt{\varepsilon_{eff}}$$

The surface reflectance *R* and absorption coefficient *μ_a_* are computed from *n_eff_* and *k_eff_* (Equations (14) and (15)).

(14) $$R=\frac{{\left(n_{eff}-1\right)}^{2}+k_{eff}^{2}}{{\left(n_{eff}+1\right)}^{2}+k_{eff}^{2}}$$

(15) $${\;\mu }_{a}=\frac{4\pi \;k_{eff}}{\lambda }$$

Particle-scale scattering is incorporated through Mie theory by computing scattering and extinction efficiencies *q_sca_* and *q_ext_* for spheres of radius *r* and complex refractive index *(n, k).* These are mapped to macroscopic coefficients via the particle number density *ρ* (Equations (16) and (18)), where *g* is the anisotropy factor.

(16) $$\mu_{s}=\rho \;q_{sca}\;\pi r^{2}$$

(17) $$\mu_{a}=\rho \left(q_{ext}-q_{sca}\right)\pi r^{2}$$

(18) $$\mu_{tr}=\mu_{a}+(1-g)\mu_{s}$$

Here, *μ_tr_* denotes the transport coefficient, where *μ_a_* is the absorption coefficient, *μ_s_* is the scattering coefficient and *g* is the Henyey–Greenstein scattering asymmetry factor (i.e., forward-scattering bias), not crystalline/material anisotropy. This yields an effective volumetric attenuation model that is compatible with Beer–Lambert absorption in the inhomogeneous coating.

Both *R* and *μ_a_* are allowed to vary with temperature using bounded affine ‘clip’ relations (Equations (19) and (20)) to capture first-order changes in optical coupling during rapid heating [13,14,15,16].

(19) $$R(T)=clip(R_{0}+a_{R}(T-T_{amb}),\;R_{min},\;R_{max})$$

(20) $${µ}_{a}(T)=clip({µ}_{a0}\;[1+a_{µ}(T-T_{amb})],\;{µ}_{min},\;{µ}_{max})$$

where $clip(x,\;x_{min},\;x_{max})=min(x_{max},\;max(x_{min},x))$

The coefficients *a_R_* and *a_μ_* are empirical temperature sensitivities used to capture first-order trends of reflectance and effective attenuation with temperature; their values and tested ranges are reported in Table 1 and treated as calibrated/assumption parameters where direct material data at 1064 nm are unavailable.

The volumetric heat source *Q(x,y,z)* is then defined such that its depth integral recovers the absorbed surface flux *(1 − R) q*_0_*(x,y)* over the coating thickness *L_z_* (Equations (21) and (22)).

(21) $$Q\left(x,y,z\right)=\left(1-R\left(T\right)\right)q_{0}\left(x,y\right)\frac{\mu_{a}e^{-\mu_{a}z}}{1-e^{-\mu_{a}L_{z}}}$$

which ensures

(22) $$\int_{0}^{Lz}Q\left(x,y,z\right)d_{z}=\left(1-R\left(T\right)\right)q_{0}(x,y)\;$$

### 2.6. Scan Kinematics and Raster

A Gaussian beam of radius *w*_0_ is scanned at speed *v_scan_* with repetition rate *f*. The incident fluence field *F_inc_(x,y)* is computed by summing the Gaussian contribution of each pulse along the programmed toolpath. Unlike an idealised constant-velocity raster, the implementation accounts for scanner dynamics through local acceleration and corner dwell times, which can create non-uniform energy delivery near turning points (Equations (23) and (24)).

To account for field-dependent focusing and morphology-induced defocus, an optional sensitivity mode allows *w*_0_ to vary spatially within a prescribed tolerance; the qualitative conclusions reported in Section 3 are robust to this perturbation [31].

(23) $$E_{p}=\frac{P_{avg}}{f}$$

(24) $${\;F}_{0}=\frac{2E_{p}}{\pi w_{0}^{2}}$$

We explicitly track pulse overlap *PO* (along-scan), line overlap *LO* (between adjacent hatch lines), and a thermal accumulation index *χ*, defined from the ratio of diffusion length to inter-pulse spatial shift (Equations (25)–(27)).

(25) $$PO=max(0,\min\left(1,\;1-\frac{v}{2w_{0}f}\right))$$

(26) $$LO=max(0,\min\left(1,\;1-\frac{s}{2w_{0}}\right))$$

(27) $$\chi =\frac{\sqrt{\frac{4\alpha }{f}}}{\frac{v_{scan}}{f}}=\frac{\sqrt{4\alpha f}}{v_{scan}}$$

Together, *PO*, *LO*, and *χ* quantify how densely pulses are packed in space and time and therefore how much cumulative heating is expected locally.

### 2.7. Incubation and Plume Shielding

The local ablation threshold *F_th_* evolves with pulse count *N_p_* through incubation (Equation (28)) [17,18]. We use a standard multi-pulse incubation law, in which *F_th_ (N_p_)* decays from an initial single-pulse threshold toward an asymptotic value as *N_p_* increases. This captures the experimentally observed reduction in effective threshold under repeated irradiation.

(28) $$F_{th}\left(N_{p}\right)=F_{\infty }+\left(F_{1}-F_{\infty }\right){N_{p}}^{-\xi }$$

The parameter *ξ* is the incubation exponent controlling the rate at which the effective threshold approaches its asymptotic value as a function of accumulated pulse count.

In parallel, the incident fluence is dynamically attenuated by ejecta and nascent plasma (“plume/plasma shielding”). We represent shielding by a dimensionless state variable *S(t)*, 0 < *S* ≤ 1, where *S* = 1 denotes no attenuation, and define the effective fluence (Equation (29)) used in the removal law as:


(29) $$F_{\mathrm{eff}}={\mathrm{S(t) F}}_{\mathrm{inc}}$$

In reduced-order form, shielding is updated pulse-to-pulse: immediately after strong material removal, the shielding strength increases (*S* decreases), and between pulses, *S* relaxes back toward unity with a characteristic plume relaxation time *τ_sh_*. This formulation (Equations (30) and (31)) is introduced as a practical proxy for plume attenuation, reported in closely spaced multi-pulse ablation at a high repetition rate [19,20,22,23].

(30) $$s_{sh}^{\left(n+1\right)}=s_{sh}^{\left(n\right)}\exp\left(-\frac{\Delta t}{\tau_{sh}}\right)+s_{max\;}[1-exp(-\kappa_{sh}\frac{D_{k}}{D_{ref}})]$$

(31) $$S^{n+1}=1-s_{sh}^{n+1},\;0<S\;\leq \;1,0\;\leq \;smax<1$$

Here, *S_sh_* is the shielding strength, *τ_sh_* is an effective plume relaxation time, *κ_sh_* controls sensitivity to removal intensity, and *D_ref_* is a reference depth increment (set to *D_cap_* unless stated otherwise).

### 2.8. Multi-Regime Ablation Model

Plume/plasma shielding is treated here as a reduced-order attenuation factor that captures the net loss of incident fluence due to absorption and scattering in the transient ablation plume above the surface. The model does not resolve the full hydrodynamic expansion of the plume; instead, *S(t)* evolves on an effective plume-lifetime timescale and is updated pulse-to-pulse, using the local repetition period *(1/f).* Spatial variability enters through the scan kinematics: the time history *S(t)* is evaluated along the moving beam position and mapped to the corresponding points *(x,y)* when constructing the effective-fluence field *F_eff_ (x,y)*. This approach provides a practical proxy for nanosecond processing, where plume dynamics occur on sub-microsecond to microsecond timescales and can significantly affect energy coupling at high repetition rates.

For each pulse *k*, we assign a depth increment *D_k_* based on *F_eff_, k* and the current *F_th_ (N_p_).* The removal law is piecewise (Equation (32)) and covers four regimes: (i) no removal (below melt onset); (ii) melt-only removal, representing viscous ejection/displacement of a thin molten layer without full vaporisation; (iii) logarithmic ablation above threshold; and (iv) blow-off at very high fluence followed by saturation beyond a cap.

(32) $$D_{k}=\left\{\begin{array}{c} 0,\;{\;F}_{eff,k}<F_{m}\\ \alpha_{m}(F_{eff,k}-F_{m}),\;F_{m}\leq F_{eff,k}<F_{th}(N_{p})\\ \mu_{a}^{-1}ln\;\frac{F_{eff,k}}{F_{th\left(N_{p}\right)}},\;F_{th}\left(N_{p}\right)\leq F_{eff},k<F_{v}\\ \mu_{a}^{-1}\ln\frac{F_{v}}{F_{th\left(N_{p}\right)}},\;F_{eff,k}\geq F_{v} \end{array}\right.$$

Regime thresholds are defined in terms of multiples of *F_th_(N_p_)* (Equations (33) and (34)).

(33) $$F_{m}=f_{m}\;F_{th}(N_{p})$$

(34) $$F_{v}=f_{v}\;F_{th}(N_{p})$$

with 0 < *f_m_* < 1 and *f_v_* > 1.

Physical bounds 0 ≤ *D_k_* ≤ *D_cap_* and *D ≤ L_z_* are enforced. The total ablation depth map is obtained by summing per-pulse increments over all pulses, contributing to each (x,y) location.

### 2.9. Thermal Calculation (One-Dimensional Solution in z, Crank-Nicolson) with Radiative-Convective Boundary Conditions

For each *(x,y)* pixel, the through-thickness problem is integrated using the transient one-dimensional heat equation (Equation (35)) with a volumetric source term *Q(x,y,z,t)* defined by Beer–Lambert absorption:

(35) $$\rho_{c}(T)\partial_{t}T(z,t)=\partial_{z}(k(T)\partial_{z}T)\;+\;Q(x,y,z,t)$$

Temperature-dependent material properties are represented using linear approximations (Equations (36) and (37)).

(36) $$k(T)=k_{0}+a_{k}(T-T_{amb})$$

(37) $$c(T)=c_{0}+a_{c}(T-T_{amb})$$

Latent-heat effects and melt/softening constraints are incorporated through additional source terms when *T* crosses the relevant thresholds (Equations (38)–(40)).

(38) $$\left(I-\sigma \partial_{zz}\right)T^{n+1}=\left(I+\sigma \partial_{zz}\right)T^{n}+\frac{\Delta t}{\rho c}Q^{n+\frac{1}{2}}$$

(39) $$\sigma =\frac{\alpha \Delta t}{2\Delta z^{2}}$$

(40) $$\alpha =\frac{k}{\rho_{c}}$$

The surface boundary condition at *z = 0* combines radiative and convective heat loss and optionally includes an evaporative sink above the boiling/sublimation threshold (Equation (41)).

(41) $${-k\partial_{z}T|}_{z=0}=h\left(T_{s}-T_{amb}\right)+\varepsilon \sigma_{SB}(T_{s}^{4}-T_{amb}^{4})$$

At *z = L_z_* (coating/substrate interface), we impose either continuity of heat flux or an effective finite-conductance/fixed-temperature constraint (Equation (42)).

(42) $$T_{s}^{n+1}=\frac{T_{s}^{*}+\frac{\Delta t}{\rho c\left(T_{s}\right)}\frac{h+4\varepsilon \sigma_{SB}T_{amb}^{3}}{\Delta z}T_{amb}}{1+\frac{\Delta t}{\rho c\left(T_{s}\right)}\frac{h+4{\varepsilon \sigma }_{SB}T_{amb}^{3}}{\Delta z}}$$

The governing equation is advanced in time using an implicit Crank–Nicolson finite-difference scheme, which is unconditionally stable for linear conduction and commonly used in laser-heating simulations with moderately temperature-dependent properties. The pulse-train envelope is normalised such that the integrated source recovers the absorbed fluence used by the thermal tier (Equation (43)).

(43) $$\sum_{n=1}^{N_{t}}Q(x,y,z,t_{n})\Delta t=\left(1-R\right)F_{eff}\;(x,y)\frac{\mu_{a}e^{-\mu_{a}z}}{1-e^{{{-\mu }_{a}L}_{z}}}$$

From the computed *T (z,t)* history, we extract the peak surface temperature, the peak subsurface temperature, and the transient melt-layer thickness *h_melt_ (x,y)*, defined as the depth range where *T* exceeds the matrix softening/melting temperature for a minimum dwell time.

The use of a reduced 1D conduction model is justified by the thin-layer geometry and the need for computational efficiency in raster-scale simulations; nevertheless, scale effects and model limits must be acknowledged. Recent heat-transfer studies on laser processing highlight how characteristic length scales affect the transient conduction and apparent thermal response, supporting the need to explicitly state modelling assumptions and their validity range [32].

### 2.10. Thin-Film Melt Hydrodynamics (Lubrication)

Given the melt-thickness map *h*_0_*(x,y)* from Section 2.9, a lubrication model advances the molten surface height *h(x,y,t)* under capillary pressure and viscous resistance (Equations (44) and (45)), with a temperature-dependent viscosity *μ(T)* (Equation (46)). This thin-film formulation is the standard long-wavelength reduction in the Navier–Stokes equations for a thin, highly viscous molten polymer layer [27,28,29]. It captures capillary-driven rim levelling and ridge broadening during the short time window before the layer re-solidifies.

(44) $${\;\partial }_{t}h=-𝛻\cdot \left(\frac{h^{3}}{3\mu \left(T\right)}𝛻p\right)$$

(45) $$p=-\gamma {𝛻}^{2}h$$

(46) $$\mu \left(T\right)=\mu_{0}\;exp(-\beta_{\mu }\left(T-T_{m}\right))$$

A diagnostic post-reflow topography *z(x,y)* is then reported (Equation (47)) to represent the frozen-in surface after capillary smoothing.

(47) $$z(x,y)\approx -D(x,y)+(h(x,y)-h_{0}(x,y))$$

### 2.11. Thermoelastic Surrogate with Confinement κ

Surface stresses are estimated using a confinement-weighted von Mises surrogate (Equation (49)), driven by the surface temperature rise *ΔT(x,y)* (Equation (48)).

(48) $$\Delta T(x,y)=T_{max}(x,y,z=0)-T_{amb}$$

This provides a first-order indicator of where thermal expansion is mechanically constrained by the surrounding, cooler material.

(49) $${\;\sigma }_{\mathrm{VM}}\left(x,y\right)=\kappa \frac{E}{1-\nu }\alpha \Delta T\left(x,y\right),\;\kappa \in \left[0,1\right]$$

In-plane displacements *u(x,y)* are obtained by solving a periodic Poisson problem (Equations (50) and (51)).

(50) $${𝛻}^{2}\;\psi =\kappa \;\alpha \;\Delta T$$

(51) u=𝛻 ψ

Here, *κ* is an empirical confinement factor (0 *≤ κ ≤* 1), and *α* denotes an effective coefficient of thermal expansion; the elastic constants *E* and *ν* are taken as effective values for the composite. This surrogate yields qualitative strain localisation and stress concentration without requiring a full thermo-mechanical finite element solution.

### 2.12. Numerical Implementation and Verification

Tier 1 (ablation) is evaluated on a uniform Cartesian grid of size L_x_ × L_y_, with spatial steps Δx_grid_ = Δy_grid_ (Table 1). The raster scan is discretised into pulses using the repetition rate f_rep_ and scan speed v_scan_; the local pulse count *N_p_*(*x, y*) is accumulated by evaluating the Gaussian fluence footprint per pulse. Dynamic shielding is implemented as a relaxation ODE, driven by local pulse history.

Tier 2 (thermal) solves the 1D transient heat equation in the coating thickness direction *z ∈* [*0, L_z_*], using a Crank–Nicolson scheme with *N_z_* nodes and *N_t_* time steps over the local exposure time; the surface boundary uses a Robin condition, combining convection and linearised radiation, and the bottom boundary is treated as adiabatic over the simulated time window.

Tier 3 (reflow) integrates a thin-film capillary-levelling equation using an explicit time-marching scheme with a stability-limited time step. To ensure numerical robustness, we performed (i) grid refinement checks (*dx*, *dy* halved) for Tier 1 depth statistics and (ii) time-step sensitivity checks for Tier 2 peak temperature and softened-layer thickness; the reported conclusions are unchanged within a small tolerance.

The Poisson problem for the displacement potential *ψ* was solved in Fourier space using an FFT-based spectral solver under periodic boundary conditions. For the thin-film reflow tier, an explicit scheme was used with a time step limited by the standard capillary-levelling stability constraint; the reported reflow results were verified to be insensitive to a further two-fold reduction in *Δt*.

## 3. Results

### 3.1. Baseline Predictions and the Impact of Scan History and Dynamic Shielding

The baseline model predicts a non-uniform depth field across the processed 2 × 2 mm window, due to the raster scan history and local boundary effects at the turnaround regions. In the baseline case, the cumulative depth map exhibits a stripe-like periodicity that follows the hatch spacing and scan direction. The corresponding regime map indicates that the processing window is dominated by the melting regime with local transitions to logarithmic ablation and, near the most overexposed regions, sporadic onset of stronger removal regimes (depending on the local fluence history).

**Figure 1:**
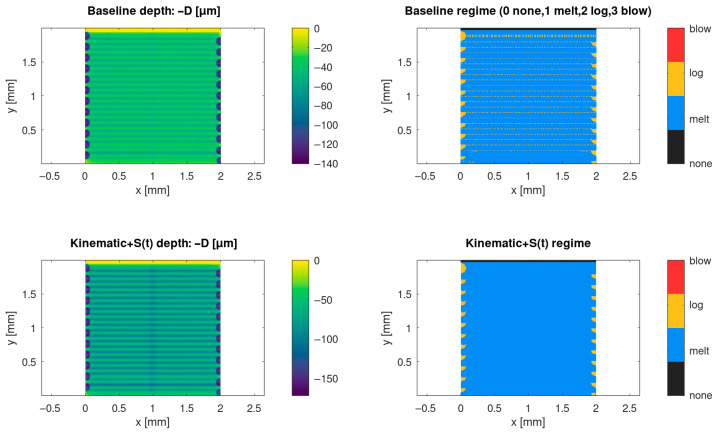
Baseline versus kinematics + dynamic shielding *S(t)*: predicted depth and regime maps. Top row: Baseline kinematic solver with simplified plume treatment. Bottom row: Kinematics-aware delivery coupled with a time-dependent shielding factor *S(t)*. Left: cumulative depth map *D(x,y)*. Right: regime map (none/melt/log/blow). The comparison isolates the impact of scan history and shielding dynamics on depth non-uniformity and regime switching.

When scan kinematics are coupled with a time-dependent shielding factor *S(t)*, the predicted depth and regime fields change appreciably relative to the baseline, reflecting the coupled effect of local dwell/acceleration and transient attenuation. In particular, the kinematics + *S(t)* formulation modifies edge exposure and the spatial pattern of regime switching driven by the scan history and shielding state, which in turn alters the depth statistics reported in Table 2. Figure 1 summarises this comparison by showing both the depth field and regime classification for the baseline and kinematics + *S(t)* cases.

### 3.2. Quantitative Cross-Case Comparison Metrics

To complement the map-based interpretation, Table 2 reports a compact set of quantitative metrics for representative cases: (i) baseline with filler (*V_f_* = 0.20), (ii) kinematics + *S(t)* with filler, and (iii) kinematics + *S(t*) without filler *(V_f_ = 0)*. The metrics include mean depth, dispersion descriptors (standard deviation and coefficient of variation), depth percentiles (*P05*, *P50*, *P95*), and the uniformity index (*UI*). This summary enables an explicit cross-case comparison without relying solely on visual inspection of maps.

Across cases, the kinematics + *S(t)* formulation changes both the central tendency (mean depth) and the spread of depth (percentiles and dispersion), indicating that transient shielding and scan history are not secondary details but primary determinants of homogeneity. The no-filler case further shows that modifying optical transport (via removal of filler-induced scattering) changes the effective coupling and therefore the resulting depth statistics under otherwise identical scan delivery and laser settings. These trends are quantitatively documented in Table 2 and are discussed mechanistically in Section 4.

Numerically, the baseline case yields *meanD* = 45 µm with *P95* = 58.28 µm and *UI* = 0.69, whereas the kinematics + *S(t)* case shifts the distribution to *meanD* = 76.25 µm with *P95* = 100.92 µm at a comparable *UI* = 0.70. Removing filler-induced optical transport (*V_f_ = 0*) yields *meanD* = 41.75 µm and *P95* = 53.58 µm, confirming that the filler representation measurably alters coupling under otherwise identical nominal settings (Table 2).

### 3.3. Parametric Sweep: Process Window Trends in the (P, v) Space

**Figure 2:**
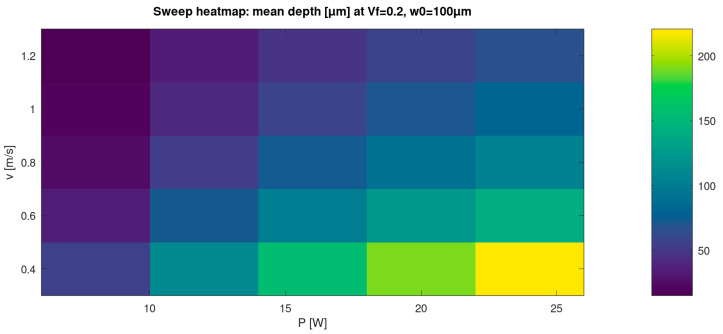
Parametric sweep map: mean ablation depth as a function of average power P and scan speed v (V_f_ = 0.20, w_0_ = 100 µm). The heatmap summarises model-predicted sensitivity of mean depth to raster parameters and provides a quantitative basis for process-window screening.

To demonstrate predictive capability beyond single-case illustrations, a parameter sweep was performed over average power P and scan speed v at fixed filler fraction (V_f_ = 0.20) and spot radius (w_0_ = 100 µm). Figure 2 presents the sweep as a heatmap of the mean depth. The response surface is strongly structured: increasing P increases the mean depth, while increasing v generally decreases it, reflecting the reduction in local dwell and accumulated fluence per unit area as the scan speed rises.

**Figure 3:**
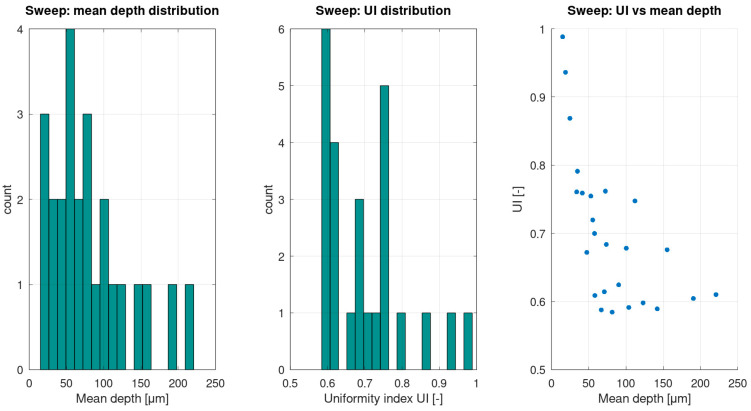
Quantitative distributions over the sweep cases: mean depth, uniformity index (*UI*), and *UI-depth* relation. Histograms summarise the spread of outcomes across the parameter space and the scatter plot reveals how uniformity varies with achieved depth.

While the heatmap provides a global view, quantitative distributions across the sweep cases provide additional insight into robustness and manufacturability. Figure 3 summarises the distribution of mean depth outcomes, the distribution of *UI*, and the relationship between *UI* and mean depth across the sweep grid. This representation directly addresses homogeneity (not only depth), showing how uniformity changes across the achievable depth range, therefore allowing for an informed selection of a process window that balances depth targeting with spatial consistency.

For transparency and reproducibility, the full sweep table (all *P-v* combinations and their metrics) is provided as Table 3. This allows readers to verify individual sweep points beyond the aggregated visualisations presented in Figure 2 and Figure 3.

### 3.4. Thermal-Tier Outputs: Absorbed Fluence, Melt-Layer Thickness, and Peak Surface Temperature

**Figure 4:**
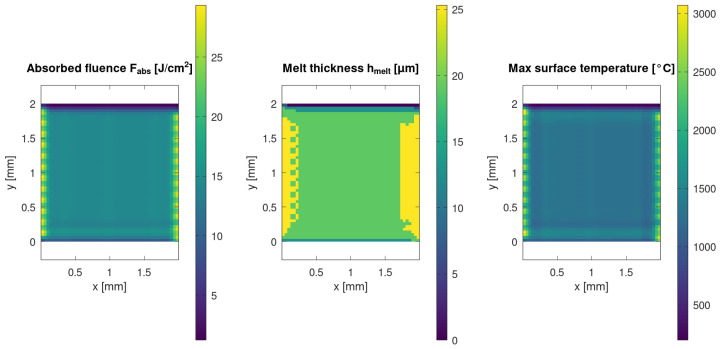
Thermal tier outputs (1D through-thickness conduction): absorbed fluence *F_abs_(x,y)*, melt-layer thickness *h_melt_(x,y)*, and peak surface temperature *T_surf_max_(x,y).* The thermal module is evaluated using the same scan delivery and shielding history as in the ablation tier, yielding spatially resolved melt-layer formation used as the initial condition for the reflow model.

The thermal tier converts the predicted absorbed energy field into a transient temperature response and melt-layer formation, using a 1D through-thickness conduction model evaluated consistently with the scan delivery history. Figure 4 presents spatial maps of absorbed fluence *F_abs_(x,y)*, melt-layer thickness *h_melt_(x, y)*, and peak surface temperature *T_surf_max_(x, y).* The maps demonstrate that melt formation is not uniform over the window and that the predicted melt-layer thickness follows the spatial structure of energy deposition and scan history.

In this framework, the thermal outputs serve two roles: (i) they provide quantitative indicators of the thermal load imposed by a given process window and (ii) they define the initial condition for the capillary reflow step. Therefore, the melt-layer thickness metric (e.g., *h_melt_*, *P95)* is included in the quantitative summary (Table 2) to facilitate cross-case comparison of the thermally activated surface mobility expected prior to reflow.

### 3.5. Capillary Reflow: Predicted Smoothing and Profile Redistribution

**Figure 5:**
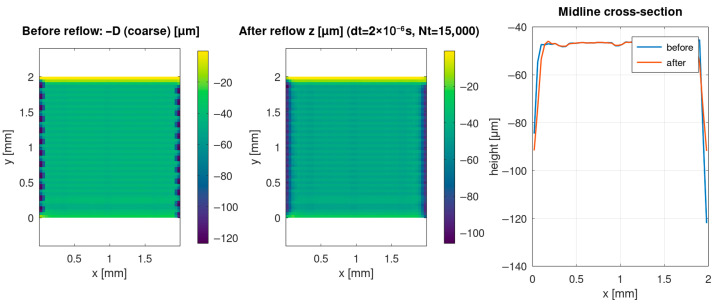
Predicted capillary reflow of the softened layer: surface topography before and after the thin-film reflow step. Maps show the coarse post-ablation surface and the reflow-modified surface; the midline cross-section highlights rim broadening and partial smoothing of the groove floor while preserving the overall depth scale.

The reflow tier models the capillary-driven redistribution of the softened layer, which modifies the post-ablation topography. Figure 5 compares the surface state before and after the reflow step, along with a midline cross-section. The results show that reflow can partially smooth high-frequency roughness and redistribute material near rims while preserving the overall depth scale set by the ablation tier. The midline comparison provides a direct, interpretable profile-level view of how the groove shape changes due to melt mobility.

These outcomes indicate that even when the ablation tier determines the gross removal depth, the final morphology that is relevant for subsequent functionalisation (e.g., selective metallisation or wettability tuning) depends on the melt-layer thickness and the ensuing reflow dynamics.

### 3.6. Effect of Filler: With-Filler Versus no-Filler Response Under Identical Settings

**Figure 6:**
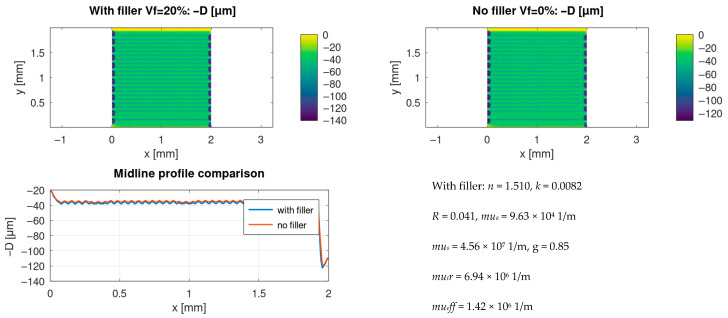
Effect of microsphere filler on predicted depth field under identical laser settings: *V_f_* = 0.20 versus *V_f_* = 0.00. Using the same scan parameters, the filler-modified optical transport model changes the effective coupling and the resulting depth statistics; the midline profile compares representative cross-sections.

To isolate the role of filler-induced optical transport, a matched comparison was performed between a composite with microsphere filler (*V_f_ =* 0.20) and the corresponding no-filler polymer case (*V_f_ =* 0) under identical laser and scan parameters. Figure 6 compares the resulting depth fields and a representative midline profile. The comparison indicates that the filler changes the effective coupling through scattering/transport proxies and thereby modifies both the spatial structure and statistics of the depth field.

Quantitatively, the same trend is reflected in Table 2 by the difference between the with-filler and no-filler kinematics + *S(t)* cases, demonstrating that filler fraction is an independent lever for tailoring the process outcome, even at fixed nominal laser power and scan speed.

## 4. Discussion

### 4.1. Mechanistic Interpretation and Process Levers

Across the three modelling tiers, a consistent mechanistic picture emerges. In the baseline case (FFT/spatially averaged plume treatment), raster scanning produces a regular groove-rim lattice whose pitch follows the programmed hatch spacing, as expected for a kinematics-idealised overlap-controlled regime (Figure 1). The corresponding regime classification is governed primarily by the local fluence history, relative to the incubation-modified threshold, with the dominant response occurring in the melting and logarithmic-ablation regimes and only limited occurrence of extreme removal (blow-off) under the baseline settings (Figure 1; Table 2) if regime fractions are reported. Under these assumptions, morphology is determined mainly by nominal overlap, duty factor, and the Gaussian beam footprint, while stochastic filler placement enters only through effective transport parameters, rather than explicit particle-resolved shadowing.

Introducing time-accurate scan delivery together with a dynamic plume/plasma shielding state *S(t)* changes this behaviour. When local acceleration/deceleration, turnarounds, and end-of-line dwell are included, and when *S(t)* is allowed to decrease after strong removal events and recover with a characteristic relaxation time, the predicted depth field develops line-to-line corrugation and intra-line modulation (Figure 1). Under otherwise identical nominal settings, deeper segments emerge near slowdowns due to an increased local dose, whereas shallower segments occur in recently attenuated regions where *S(t)* remains below unity. The resulting depth distributions broaden and can become multi-modal (Figure 3, Table 2), which is consistent with recurring combinations of scan-delivery state (steady motion versus turnarounds) and shielding state (unattenuated versus recently attenuated). This indicates that discrete depth “levels” arise from coupled scan-shielding dynamics, rather than from numerical artefacts. Practically, this implies that predictive process-window mapping for high-repetition-rate scanning should account for realistic scan kinematics and transient attenuation, not solely average power and nominal spot size (Figure 2 and Figure 3, Table 3).

Enabling the thermal tier introduces temperature-dependent optical coupling and one-dimensional (through-thickness) heat diffusion while preserving the scan-delivery and shielding history. This yields spatially structured absorbed-energy and melt-formation fields and predicts a continuous molten layer along portions of the scan tracks under representative conditions (Figure 4). From the transient temperature history, the melt-layer thickness *h_melt_* is extracted as the depth range over which the matrix exceeds the softening/melting threshold for a minimum dwell time. In the representative thermal case, *h_melt_* reaches the order of tens of micrometres, which provides the initial condition for the thin-film (lubrication) reflow step and rim relaxation (Figure 4 and Figure 5, Table 2). In addition, local melting and material displacement may promote exposure of PDA/Ag-coated microspheres at the surface, which is relevant for subsequent selective electroless metallisation, as well as wetting and adhesion control in filled polymer coatings.

Taken together, the tiers quantify three practical levers. First, overlap and hatch geometry set the nominal groove pitch and depth in the kinematics-idealised, incubation-governed regime. Second, realistic scan kinematics and the shielding history *S(t)* control long-wavelength corrugation, intra-line modulation, and the emergence of discrete depth modes in statistical distributions. Third, the melt-layer thickness predicted by the thermal tier governs (i) the extent of capillary smoothing captured by the thin-film reflow step and (ii) the likelihood of filler exposure at the surface, thereby defining the initial condition for downstream metallisation, bonding, or surface-functionalisation steps.

The present framework is deliberately reduced-order. Thermal transport is solved in one dimension, rather than fully in three dimensions; melt redistribution is represented using a thin-film (lubrication) model instead of a full free-surface Navier–Stokes treatment; and stress is estimated via a confinement-weighted thermoelastic surrogate, rather than a fully coupled thermomechanical solution. Plume/plasma shielding is represented as a single state variable with a relaxation time, rather than by resolving plume expansion and radiative/absorptive transport. Moreover, no direct experimental measurements for this PLA/PDA/Ag-filled system are reported here, and the model has not been quantitatively calibrated against profilometry, cross-section microscopy, or melt-layer thickness mapping. These simplifications keep the workflow computationally lightweight and suitable for rapid process-window exploration, while also defining the limits of quantitative interpretation.

Future work should calibrate incubation parameters, plume relaxation time, viscosity–temperature relationships, and *h_melt_* predictions against measured depth profiles, rim widths, and melt-layer thicknesses obtained from profilometry and microscopy. Extending the thermal tier to include lateral heat diffusion and adopting more complete free-surface flow formulations would improve the quantitative prediction of post-process roughness and rim rounding. Finally, expanding the material-property inputs (optical constants at 1064 nm, thermal diffusivity, and viscosity as a function of temperature) would support the transfer of the workflow to other filled polymers and coatings under industrial scan speeds and raster strategies.

### 4.2. Literature-Based Qualitative Validation and Limitations

The present work is numerical; therefore, direct experimental validation is not included in this manuscript. To nevertheless assess plausibility, we compare the predicted trends with published observations of nanosecond laser raster processing of industrial polymers. Prior studies report that the polymer response under nanosecond irradiation depends strongly on scan overlap and accumulated pulse count, and that non-uniform grooves can arise when scanner dynamics and local dwell are not controlled [33]. Our model reproduces these qualitative dependencies: depth non-uniformity increases near turnaround regions and overlap-dominated zones, and dynamic shielding modifies the effective delivered fluence under identical average-power settings (Figure 1, Figure 2 and Figure 3).

Reported nanosecond/NIR laser micromachining of thermoplastics under raster-like strategies yields groove/channel depths that are typically on the order of tens to ~10^2^ µm, with the achievable range extending beyond 10^2^ µm, depending on fluence, overlap and dwell conditions. In the present simulations, representative outcomes span *P95* ≈ 54–101 µm for the cross-case comparisons (Table 2), while the broader (*P, v*) sweep reaches mean depths up to ~221 µm (Table 3). These magnitudes are consistent with published polymer micromachining depth ranges and support plausibility prior to direct experimental calibration.

A key limitation is that the thermal tier is reduced (1D, pulse-averaged) to enable raster-scale simulation; scale-dependent transient conduction can influence the apparent thermal response, and the model’s validity range should be interpreted accordingly [32,34]. Future work will integrate profilometry and microscopy topographies for calibration of effective optical and ablation parameters and to validate the predicted depth statistics, melt-layer thickness, and post-reflow morphology.

## 5. Conclusions

(i) The Tier 1 ablation module predicts cumulative depth fields whose statistics and spatial uniformity depend not only on nominal laser settings but also on scan history effects, including pulse overlap and local dwell during raster turnarounds (Table 2; Figure 1, Figure 2 and Figure 3).
(ii) Incorporating scan-kinematics-aware pulse accumulation with a time-dependent plume/plasma shielding factor *S(t)* shifts both depth statistics and uniformity metrics, indicating that kinematics and transient attenuation can dominate outcomes beyond average power alone (Table 2).
(iii) The filler-enabled optical transport representation modifies effective coupling under identical nominal settings, measurably affecting depth distributions and regime occurrence, thereby clarifying the role of microspheres within the proposed workflow (Table 2; Figure 6).
(iv) Coupling the thermal and reflow tiers provides melt-layer and morphology-redistribution descriptors that complement ablation-depth predictions, enabling quantitative process-window screening prior to experimental calibration (Figure 4 and Figure 5).

This work presents a reduced-order, tiered numerical workflow for pulsed-laser surface processing of a particle-filled polymer coating under high-repetition-rate, nanosecond-class 1064 nm irradiation. The workflow couples scan-path delivery (kinematics), incubation-driven threshold evolution, and plume/plasma shielding to predict cumulative ablation depth; it also extends the framework with temperature-dependent optical coupling and one-dimensional (through-thickness) heat diffusion to estimate the melt-layer thickness *h_melt_* and applies a thin-film (lubrication) reflow step to approximate post-process rim morphology and groove smoothing.

Three practical outcomes follow.

(1) Under kinematics-idealised constant-velocity scanning with spatially averaged attenuation, the predicted morphology forms a regular groove-rim lattice whose pitch is primarily set by hatch spacing and overlap, consistently with an overlap-controlled regime.
(2) When realistic scan kinematics and time-dependent plume/plasma shielding *S(t)* are included, line-to-line corrugation and intra-line depth modulation emerge, even at fixed nominal settings. Depth distributions broaden and may become multi-modal, which is consistent with recurring scan-shielding states (steady-pass segments, turnaround dwell regions, and recently attenuated segments). This indicates that scan history and transient attenuation–not only average power and nominal spot size–govern long-wavelength non-uniformity under high-repetition-rate raster scanning.
(3) Enabling the thermal tier predicts the formation of a molten layer along portions of the scan track, providing the initial condition for thin-film capillary reflow. The reflow step broadens and lowers rims and partially smooths groove floors. In addition, local melting and material displacement may promote microsphere exposure at the surface, which is relevant for subsequent selective electroless metallisation and for wetting/adhesion control.

Collectively, the results indicate that overlap and hatch geometry, scan kinematics, plume/plasma shielding history *S(t)*, and melt-layer thickness *h_melt_* constitute a compact set of controllable levers for engineering groove depth, rim width, corrugation amplitude, and the likelihood of filler exposure in particle-loaded polymer coatings processed at industrial scan speeds.

The present study does not yet include experimental validation for the PLA/PDA/Ag-filled system. Quantitative calibration of incubation parameters, plume relaxation time, viscosity–temperature relationships, and predicted *h_mel_*_t_ against profilometry, cross-section microscopy (e.g., SEM), and melt-layer measurements is therefore required. Thermal transport is treated as one-dimensional through thickness, melt redistribution is represented using a thin-film approximation rather than full free-surface flow, and stress is estimated using a thermoelastic surrogate. These simplifications keep runtime low but define the limits of quantitative accuracy.

The workflow is portable: replacing optical constants, thermophysical properties, and viscosity–temperature data enables direct application to other filled polymers and coating systems, including materials designed for laser-activated metallisation, adhesion tuning, or functional wetting.

## Figures and Tables

**Table 1:** Numerical, laser, and material parameters used in the baseline simulations and parametric sweeps. Parameters include domain and discretisation (*L_x_*, *L_y_*, *L_z_*), laser settings (*λ*, *f_rep_*, *P_avg_*, *v_scan_*, *w*_0_, hatch, turnaround dwell), incubation and regime-switching constants (*F_1_*, *F_∞_*, *ξ*, *f_m_*, *f_v_*, *α_m_*, *D_cap_*), optical transport proxies (*V_f_*, $\bar{r}$, *σ_r_*, *μ_a_*, *μ_s_*, *g*), and thermal-module settings (*ρ*, *c_p_*, *k*, *η_heat_*, *T_m_*, *N_zth_*, *N_tth_*). Where direct material data at 1064 nm were unavailable, parameters were treated as calibrated assumptions and are explicitly marked/used only for sensitivity screening.

Parameter	Value	Units	Meaning
*L_x_*	0.002	m	Lateral domain size (x)
*L_y_*	0.002	m	Lateral domain size (y)
*L_z_*	0.0005	m	Coating thickness
*Δx_grid_*	1 × 10^−5^	m	Spatial step in x (surface grid)
*Δy_grid_*	1 × 10^−5^	m	Spatial step in y (surface grid)
*λ*	1.064 × 10^−6^	m	Laser wavelength (Nd:YAG)
*f_rep_*	60,000	Hz	Pulse repetition rate
*P_avg_*	12	W	Average laser power (baseline for mapping)
*v_scan_*	1	m/s	Scan speed (baseline)
*w*_0_	0.0001	m	Gaussian radius (1/e^2^ intensity)
*h*	8 × 10^−5^	m	Hatch spacing (line-to-line)
*t_turn_*	0.0003	s	Turnaround dwell time (end-of-line pause)
*Aw_0_*	0.05	-	Relative amplitude of positional spot-size variability
*k_defocus_*	0.4	-	Depth-dependent defocus strength (optional)
*V_f_*	0.2	-	Filler volume fraction (glass microspheres)
$\bar{r}$	2.5 × 10^−5^	m	Mean microsphere radius
*σ_r_*	6 × 10^−6^	m	Microsphere radius standard deviation
*g*	0.85	-	Henyey–Greenstein scattering asymmetry factor
*C_s_*	12	-	Effective scattering-strength proxy (dimensionless calibration parameter)
*F_1_*	8000	J/m^2^	Single-pulse ablation threshold (N = 1)
*F_∞_*	3000	J/m^2^	Asymptotic threshold for large pulse count
*ξ*	0.25	-	Incubation exponent in threshold evolution
*f_m_*	0.6	-	Melt onset multiplier (relative to local threshold)
*f_v_*	3	-	Blow-off cap multiplier (relative to local threshold)
*α_m_*	2 × 10^−11^	m/(J/m^2^)	Melt-depth coefficient in the melt regime
*D_cap_*	5 × 10^−6^	m	Per-pulse depth increment cap (numerical/physical safeguard)
*ρ*	1240	kg/m^3^	Density (PLA baseline)
*c_p_*	1800	J/kg/K	Specific heat capacity
*k*	0.13	W/m/K	Thermal conductivity
*η_heat_*	0.25	-	Absorbed-to-heat efficiency (lumped)
*N_z_*	80	-	Number of depth nodes in 1D thermal grid
*N_t_*	120	-	Number of time steps per thermal cell/block
*d_s_*	4	-	Block-mean/subcycling factor for thermal stepping
*T_m_*	453.15	K	Melt threshold temperature

**Table 2:** Summary metrics for representative simulations used for cross-case comparison. Reported are mean depth *(meanD)*, dispersion (*stdD, CV*), uniformity index (*UI*), depth percentiles (*P05*, *P50*, *P95*), melt-layer thickness metric (*h_melt_*, *P95*), and peak surface temperature (*T_surf_max_, K*) for: (i) baseline kinematic solver with filler (*V_f_ =* 0.20), (ii) kinematics + dynamic shielding S(t) with filler, and (iii) kinematics + *S(t)* without filler (*V_f_* = 0). Metrics are computed over the full 2 × 2 mm window. The reported *T_surf_max_* values originate from the reduced 1D heat-conduction tier and should be interpreted as a comparative indicator of thermal loading, rather than an absolute physical surface temperature, because polymer decomposition and associated energy sinks are not explicitly resolved in the present formulation.

Case	*V_f_*	*P*, W	*v*, m/s	*w*_0_, µm	*meanD*, µm	*stdD*, µm	*CV*
baseline	0.2	12	1	100	45	18.10	0.40
kinematic	0.2	12	0.6	100	76.254	22.86	0.30
kinematic_no_filler	0	12	1	100	41.746	16.64	0.40
Case	*UI*	*P05*, µm	*P50*, µm	*P95*, µm	*h_melt_*, *P95*, µm	*T_surf_max_,* K	
baseline	0.69	27.27	43.02	58.28	25.32	3070.68	
kinematic	0.70	47.20	75.80	100.92	31.65	3129.35	
kinematic_no_filler	0.68	25.01	39.90	53.58	25.32	2679.76	

**Table 3:** Full parameter sweep results (*P × v*) for *V_f_* = 0.20 and *w*_0_ = 100 µm. For each combination of average power P and scan speed v, the table reports mean depth, uniformity index (*UI*), melt-layer thickness metric (*h_melt_*, *P95*, µm), and peak surface temperature (*T_surf_max_*, K). This table supports quantitative trend analysis beyond figure-based inspection.

*P*, W	*v*, m/s	*V_f_*	*w*_0_, µm	*meanD*, µm	*UI*	*h_melt_*, *P95*, µm	*T_surf_max_*, K
8	0.4	0.2	100	55.45	0.71	37.97	1898.89
12	0.4	0.2	100	112.09	0.74	44.30	2744.10
16	0.4	0.2	100	155.23	0.67	44.30	3600.22
20	0.4	0.2	100	190.46	0.60	50.63	4466.68
24	0.4	0.2	100	220.78	0.61	50.63	5325.51
8	0.6	0.2	100	34.90	0.79	25.31	1532.44
12	0.6	0.2	100	72.44	0.76	31.64	2249.31
16	0.6	0.2	100	100.46	0.67	37.97	2947.97
20	0.6	0.2	100	123.01	0.59	37.97	3655.95
24	0.6	0.2	100	141.94	0.58	37.97	4367.00
8	0.8	0.2	100	24.81	0.86	18.98	1300.99
12	0.8	0.2	100	52.93	0.75	25.31	1925.23
16	0.8	0.2	100	73.64	0.68	31.64	2518.82
20	0.8	0.2	100	90.25	0.62	31.64	3125.89
24	0.8	0.2	100	103.84	0.59	31.64	3740.51
8	1	0.2	100	18.91	0.93	18.98	1154.39
12	1	0.2	100	41.44	0.75	18.98	1701.09
16	1	0.2	100	57.85	0.69	25.31	2243.55
20	1	0.2	100	71.00	0.61	25.31	2776.83
24	1	0.2	100	81.51	0.58	25.31	3317.20
8	1.2	0.2	100	15.07	0.98	12.65	1042.27
12	1.2	0.2	100	33.86	0.76	18.98	1535.81
16	1.2	0.2	100	47.46	0.67	18.98	2029.80
20	1.2	0.2	100	58.32	0.60	25.31	2515.15
24	1.2	0.2	100	66.91	0.58	25.31	3001.62

## Data Availability

The original contributions presented in this study are included in the article. Further inquiries can be directed to the corresponding authors.

## Nomenclature

Laser delivery and fluence	
*F_inc_(x,y)*	incident fluence before shielding at location (*x,y*). [J·m^−2^]
*S(t)*	plume/plasma shielding factor, 0 < *S(t)* ≤ 1; *S* = 1 denotes no shielding. [-]
*F_eff_(x,y)*	effective fluence after shielding, *F_eff_* = S(t) Finc. [J·m^−2^]
*Fth(N_p_)*	local ablation threshold after *N_p_* pulses (incubation-modified). [J·m^−2^]
*E_p_*	pulse energy, *E_p_* = *P_avg_/f_rep_*. [J]
*f_rep_*	pulse repetition rate. [Hz]
*τ_p_*	pulse duration. [s]
*v_scan_*	scan speed along the raster track. [m·s^−1^]
*w*_0_	Gaussian beam radius (*1/e*^2^). [m]
*h*	hatch spacing (line-to-line). [m]
Overlap and accumulation	
*PO/LO*	pulse overlap (along-scan)/line overlap (between hatch lines). [-]
*χ*	thermal accumulation index defined from diffusion length and inter-pulse spatial shift. [-]
*N_p_(x,y)*	number of pulses accumulated at location (*x,y*). [-]
Removal and morphology	
*D_k_(x,y)*	depth increment from pulse k at *(x,y)* (multi-regime law). [m]
*D(x,y)*	cumulative ablation depth, *D(x,y) = ∑_k_D_k_(x,y)*. [m]
*D_cap_*	per-pulse depth increment cap. [m]
Optical transport (effective medium + scattering)	
*n_eff_, k_eff_*	effective refractive index and extinction coefficient. [-]
*ε_eff_*	effective complex permittivity. [-]
*R*	surface reflectance. [-]
*μ_a_*	Beer–Lambert absorption coefficient. [m^−1^]
*μ*_s_	scattering coefficient. [m^−1^]
*μ_tr_*	transport attenuation coefficient, *μ_tr_ = μ_a_ + (1 − g)μ_s_*. [m^−1^]
*g*	Henyey–Greenstein scattering asymmetry factor. [-]
Thermal and reflow	
*T(z,t)*	transient temperature field (1D through thickness). [K]
*ΔT(x,y)*	surface temperature rise, relative to ambient. [K]
*h_melt_(x,y)*	transient melt-layer thickness. [m]
*μ(T)*	temperature-dependent melt viscosity used in the thin-film model. [Pa·s]
*γ*	surface tension in the lubrication model. [N·m^−1^]
Microstructure generation	
*N_s_*	number of spheres in the representative microstructure box. [-]
*RSA/PBC*	random sequential adsorption/periodic boundary conditions.
Materials	
*PLA*	polylactic acid (matrix).
*PDA*	polydopamine coating.
*PDA/Ag microspheres*	glass microspheres coated with PDA and Ag.
